# Factors Influencing Australian Healthcare Workers’ COVID-19 Vaccine Intentions across Settings: A Cross-Sectional Survey

**DOI:** 10.3390/vaccines10010003

**Published:** 2021-12-21

**Authors:** Jessica Kaufman, Kathleen L. Bagot, Monsurul Hoq, Julie Leask, Holly Seale, Ruby Biezen, Lena Sanci, Jo-Anne Manski-Nankervis, J. Simon Bell, Jane Munro, Carol Jos, Darren Suryawijaya Ong, Jane Oliver, Jane Tuckerman, Margie Danchin

**Affiliations:** 1 Vaccine Uptake Group, Murdoch Children’s Research Institute, Melbourne 3052, Australia; katie.bagot@mcri.edu.au (K.L.B.); monsurul.hoq@mcri.edu.au (M.H.); Jane.Munro@rch.org.au (J.M.); carol.jos@mcri.edu.au (C.J.); darren.suryawijaya@mcri.edu.au (D.S.O.); jane.oliver@mcri.edu.au (J.O.); jane.tuckerman@mcri.edu.au (J.T.); margie.danchin@rch.org.au (M.D.); 2 Department of Paediatrics, University of Melbourne, Melbourne 3052, Australia; 3 Susan Wakil School of Nursing and Midwifery, University of Sydney, Sydney 2052, Australia; julie.leask@sydney.edu.au; 4 School of Population Health, University of New South Wales, Sydney 2052, Australia; h.seale@unsw.edu.au; 5 Department of General Practice, University of Melbourne, Carlton 3010, Australia; ruby.biezen@unimelb.edu.au (R.B.); l.sanci@unimelb.edu.au (L.S.); jomn@unimelb.edu.au (J.-A.M.-N.); 6 Centre for Medicine Use and Safety, Faculty of Pharmacy and Pharmaceutical Sciences, Monash University, Melbourne 3052, Australia; simon.bell2@monash.edu; 7 The Royal Children’s Hospital, Melbourne 3052, Australia; 8 The Peter Doherty Institute for Infection and Immunity, University of Melbourne, Melbourne 3000, Australia

**Keywords:** immunization, vaccination, coronavirus, vaccine acceptance, communication

## Abstract

Healthcare workers’ COVID-19 vaccination coverage is important for staff and patient safety, workforce capacity and patient uptake. We aimed to identify COVID-19 vaccine intentions, factors associated with uptake and information needs for healthcare workers in Victoria, Australia. We administered a cross-sectional online survey to healthcare workers in hospitals, primary care and aged or disability care settings (12 February–26 March 2021). The World Health Organization Behavioural and Social Drivers of COVID-19 vaccination framework informed survey design and framing of results. Binary regression results adjusted for demographics provide risk differences between those intending and not intending to accept a COVID-19 vaccine. In total, 3074 healthcare workers completed the survey. Primary care healthcare workers reported the highest intention to accept a COVID-19 vaccine (84%, 755/898), followed by hospital-based (77%, 1396/1811) and aged care workers (67%, 243/365). A higher proportion of aged care workers were concerned about passing COVID-19 to their patients compared to those working in primary care or hospitals. Only 25% felt they had sufficient information across five vaccine topics, but those with sufficient information had higher vaccine intentions. Approximately half thought vaccines should be mandated. Despite current high vaccine rates, our results remain relevant for booster programs and future vaccination rollouts.

## 1. Introduction

Healthcare workers (HCWs) were prioritised to receive COVID-19 vaccines in most countries, due to the high occupational risk of COVID-19 infection [1,2,3,4]. Systematic reviews of the international literature report that HCW vaccine acceptance rates between countries varied between 28% and 96% (data published up to March 2021) [5,6] with hesitancy associated with vaccine safety (e.g., side effects), rapid development, and vaccine efficacy and effectiveness (data published up to February 2021) [7]. COVID-19 vaccine intentions [8,9] and vaccine uptake [10,11] also varies by clinical discipline and setting internationally. As of 26 November 2021, there have been 4092 COVID-19 cases among clinical HCWs in Victoria, Australia, with 66% of infections likely acquired in workplace [12]. Of these workplace cases, 50% have been in aged care or disability care workers and 39% in nurses, with most occurring prior to the availability of COVID-19 vaccines [12].

By 10 November 2021, 81.5% of Australians aged over 16 years have received two doses of a COVID-19 vaccine. Rates of vaccine uptake among Australian HCWs are only reported separately for aged care workers, of whom 95.8% (*n* = 252,249) were fully vaccinated (10 November 2021) [13]. National mandatory vaccination policies apply to aged care workers [14] and state policies apply for other HCWs, which vary across jurisdictions and settings [15,16]. However, there are no publicly available data on coverage for other Australian HCWs by role or workplace setting.

Our study was conducted at the beginning of the vaccine rollout in Australia in early 2021, prior to the introduction of any vaccination requirements for HCWs. We surveyed those working in hospitals, primary care and aged and disability care settings who were prioritised to receive the COVID-19 vaccine in Victoria, Australia, to understand COVID-19 vaccine intentions and the factors that influence uptake. We aimed to (1) identify vaccination intention rates by setting for frontline healthcare, and aged and disability care workers, (2) explore factors associated with vaccine hesitancy or refusal and (3) identify information needs to support receiving and recommending the COVID-19 vaccine.

## 2. Materials and Methods

### 2.1. Participants, Setting and Context

As part of a larger, mixed-methods study [17], this cross-sectional study was conducted in Victoria, Australia between 12 February and 26 March 2021 at an early stage of the Australian vaccination program rollout. Although Victoria was the epicentre for Australian COVID-19 cases during 2020 and had a 5-day “circuit breaker” lockdown between 12–17 February 2021 [18], Victoria had minimal community transmission [19] and few COVID-19 related restrictions at the time of this survey. The study was funded by the Victorian Department of Health and was conducted to support the Victorian Government’s COVID-19 vaccine communication planning.

Australia’s vaccination program commenced 22 February 2021, with Pfizer-BioNTech (BNT162b2) and AstraZeneca (Oxford, ChAdOx1) COVID-19 vaccines available to those prioritised in Phase 1a (i.e., quarantine and border workers, frontline HCW sub-groups and other HCWs, aged care and disability care staff and residents) and 1b (i.e., general public aged 70 years and over, non-frontline HCWs, Aboriginal and Torres Strait Islander people aged over 55 years, adults with an underlying condition or disability, other critical and high risk workers) [3]. Both of these vaccines were available to these prioritised groups via hospital-based and general practice (from 22 March 2021)-based vaccination programs. In January 2021, media commentary was focusing on the lower effectiveness of AstraZeneca (compared to Pfizer) [20,21] and by mid-March 2021, several European countries had suspended the use of AstraZeneca due to risks of clotting [22].

HCWs living in Victoria were eligible to complete the online survey. Eligible occupations included nurses, medical doctors, pharmacists, allied health professionals, personal support workers, ambulance staff or paramedics, and other health professionals (e.g., dentist, psychologist, etc.), working at a hospital, healthcare practice (community or private) or residential aged or disability care setting in Victoria. A combination of snowballing recruitment and research advertisements across health services, clinical colleges, councils, associations, unions, networks, and Facebook were used to recruit participants.

### 2.2. Data Collection

The survey was developed specifically for COVID-19 vaccination with between 35 and 46 items, depending on participant responses (see Table S1 for full survey). We drew 11 items from the World Health Organization Behavioural and Social Drivers of Immunisation (BeSD) COVID-19 vaccine survey that covers the domains of thinking and feeling, social processes and practical issues, as well as the motivation domain that measures vaccine intentions [23]. A further 13 items were developed by the research team, in consultation with the Victorian Government Department of Health representatives. In addition to demographics (7 items) and workforce characteristics (4 items), survey items covered vaccine intentions (i.e., ‘If a COVID-19 vaccine were recommended for you, would you get it?’ with response options ’Yes’, ‘No’, ‘Not sure’), factors participants identify as influencing their vaccination decision making, vaccine concerns and beliefs, perceived convenience of getting the vaccine and information needs. We also included items covering perceptions on mandating and recommending the vaccine to patients. Response options were categorical.

The 10-minute survey and participant information statement were administered online via REDCap [24]. The survey could be completed anonymously, and consent was implied by survey completion. The Royal Children’s Hospital Human Research Ethics Committee provided ethics approval for the mixed-methods study (HREC/72845/RCHM-2021).

### 2.3. Analysis

**Figure 1:**
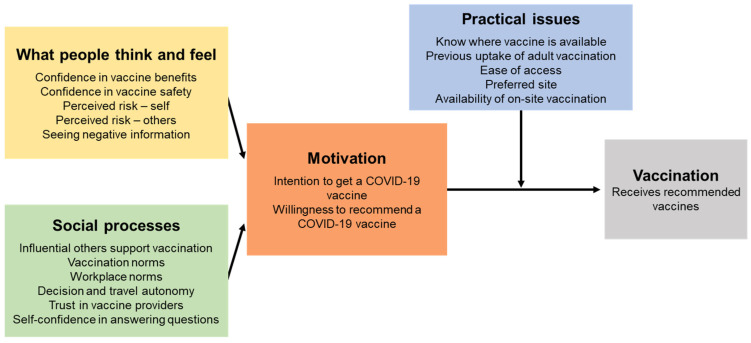
The Behavioural and Social Drivers of COVID-19 vaccination framework [25]. CC BY-NC-SA 3.0 IGO, https://www.who.int/publications/i/item/WHO-2019-nCoV-vaccination-demand-planning-2021.1 (accessed on 15 December 2021).

Participants who did not report their vaccine intention or occupational setting were excluded, as were those with unclear or uncategorisable (e.g., multiple settings) occupations settings provided. As multiple professional roles (e.g., physicians, nurses) are in each setting, results are presented for the total sample, and then by hospital, primary care and aged/disability setting. Categorical responses are presented as number and percentages. For questions supporting multiple responses, results are presented in order of frequency of selection. Binary regressions were used to estimate risk differences (RD) with 95% confidence intervals (CIs) between those intending and those unsure/not intending to accept a COVID-19 vaccine for different demographic characteristics. For comparing the intention to accept a COVID-19 vaccine based on different factors, binary regressions were used adjusted for age, sex, cultural and linguistically diverse (CALD) status (born overseas and/or speaking a language other than English at home), employment status and regionality. With missing data rates < 5% across surveys, we have analysed complete cases. Results are presented according to the BeSD framework [25] which outlines the measurable and modifiable drivers of vaccine uptake (Figure 1). Where responses to one question are relevant to more than one component of the BeSD framework, results are presented in only one section (e.g., all information results are presented in practical issues). Data were analysed using Stata 16.1 [26].

## 3. Results

In the first quarter of 2021, a total of 3224 participants completed the survey, of which 150 respondents were excluded as they did not report their vaccine intentions (*n* = 7), did not report their occupational setting (*n* = 28) or reported it as ‘other’ (*n* = 115). Demographic characteristics of the remaining 3074 participants differed across settings in some key areas (Table 1). Compared to those working in hospital or aged care settings, fewer HCWs in primary care settings were female, fewer were nurses and more were employed full time. The proportion of CALD participants and those living in regional areas was higher in aged care than in other settings. A higher proportion of HCWs in hospital settings were younger than 50 years.

### 3.1. Motivation

Primary care HCWs reported the highest intention to accept a COVID-19 vaccine (84%, 755/898), followed by hospital-based HCWs (77%, 1396/1811) and aged care workers (67%, 243/365) (Table 2). Intention to be vaccinated was higher among men, people aged 50 years or older, employed full time or living in major cities. Compared to nurses (77%, 1674/2169), the intention to be vaccinated was 17% (95% CI 13 to 21%) higher in medical doctors and 21% (95% CI 9 to 33%) lower in personal support staff.

Eighty percent of participants (80%, 2388/2981) would recommend a COVID-19 vaccine to eligible patients. Aged care workers were the least likely to recommend the vaccine (65%, 227/348) compared to hospital (80%, 1412/1759) or primary care HCWs (86%, 749/874). Of those HCWs who would not make a recommendation, only 25% (149/593) were planning to get the vaccine themselves.

### 3.2. Thinking and Feeling

#### 3.2.1. Factors Influencing COVID-19 Vaccine Decision-Making

**Figure 2:**
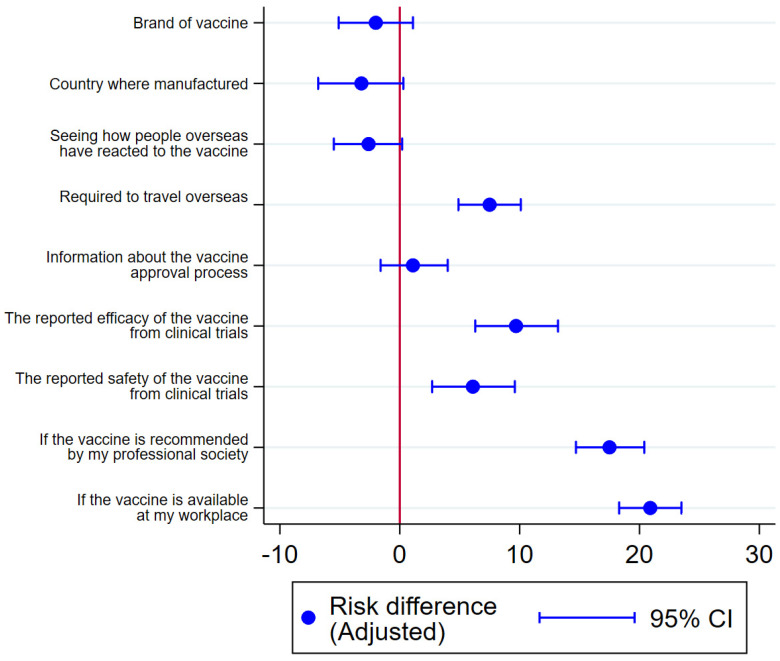
Factors associated with intention to accept a COVID-19 vaccine. Risk difference adjusted for age, sex, CALD, employment and remoteness.

The factors HCWs most commonly perceived as influencing their personal vaccine decision making were vaccine safety (73%, 2243/3074), vaccine efficacy (71%, 2170/3074) and seeing how people overseas reacted to the vaccines (56%, 1708/3074). However, the factors statistically associated with the intention to accept a vaccine were vaccine availability at the workplace and vaccine recommendation by professional society (Figure 2 and Table S2). The associations were weak between the intention to vaccinate and brand of vaccine offered, manufacturing country, seeing how people overseas reacted to the vaccine or information about the approval process for vaccination in Australia.

#### 3.2.2. Vaccine Concerns for Those Unsure or Not Intending to Accept the Vaccine

**Figure 3:**
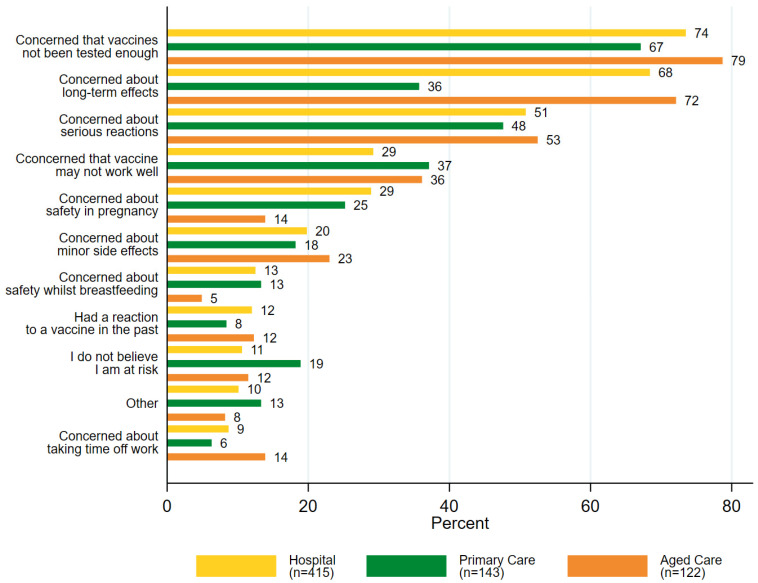
Concerns of HCWs (% selected) who were unsure or did not intend to accept a COVID-19 vaccine (*n* = 680), by setting.

Of those HCWs who were unsure or not intending to accept a COVID-19 vaccine (*n* = 680), the primary concern across all settings was the belief that the vaccines have not been tested enough for safety (73%, 497/680). While concern about potential long-term effects of the vaccine was the next most common concern in hospital (68%, 284/415) and aged care settings (72%, 88/122), primary care HCWs were more concerned about serious reactions (48%, 68/143) (Figure 3 and Table S3).

#### 3.2.3. Perceived Risks of COVID-19

Forty two percent of HCWs (42%, 1287/3069) were very or moderately concerned about getting COVID-19 themselves, with negligible difference across settings. Approximately half of all HCWs across settings were not concerned about passing COVID-19 to their patients, with those working in aged care (59%, 215/363) more concerned than those in primary care (51%, 456/895) or hospitals (49%, 891/1808) (Table S3). However, in the hospital setting, HCWs who were concerned about passing COVID-19 to their patients were more likely to accept a vaccine, compared to those who were not concerned (adjusted risk difference 9%, 95% CI 5 to 12%). There was no evidence of associations between perceived risks of COVID-19 and the intention to receive a vaccination in other settings (Table 3).

#### 3.2.4. Beliefs about COVID-19 Vaccines

Across all settings, trust in the vaccines and the belief that the vaccines were important, safe, would protect others and would not cause a serious reaction were strongly associated with the intention to vaccinate (Table 3). HCWs in primary care settings had the highest rate of agreement across these COVID-19 vaccine beliefs, while those working in aged care had the lowest (Table S3).

### 3.3. Practical Issues

#### 3.3.1. Information about COVID-19 Vaccines

Only 25% (782/3074) of participants felt they had enough information about all five of the following COVID-19 vaccine topics: how the COVID-19 vaccines work, vaccine effectiveness, vaccine safety, vaccine side effects and vaccine dose and interval recommendations (Table S3). Those working in aged care settings felt the least informed about each topic. Compared to HCWs across settings who did not feel informed about at least one topic, HCWs who felt sufficiently informed about all five topics had higher vaccine intentions; after adjusting for demographics, vaccine intentions were 20% higher for hospital HCWs (95% CI 17 to 22%), 13% higher for primary care HCWs (95% CI 9 to 17%) and 32% higher in aged care (95% CI 25 to 39%) (Table 3).

#### 3.3.2. Perceived Convenience of COVID-19 Vaccination

Most participants (82%, 2503/3056) thought it would be moderately or very convenient to get a COVID-19 vaccine. Fewer of those working in aged care settings thought it would be convenient (76%, 276/362) compared to those working in primary care (80%, 708/894) or hospital settings (84%, 1519/1800). Across all settings, the primary concern among those who thought it would not be very convenient to get the vaccine was organising an appointment at a suitable time (45%, 758/1703). For those in aged care settings, the next most common concern was travelling to the vaccine location (28%, 63/229). However, the second most common concern for those in the hospital setting was knowing which vaccine priority group they were in (33%, 297/903), and for primary care HCWs it was long wait times (41%, 231/571). Intention to vaccinate was significantly higher among HCWs who thought it would be convenient to receive a COVID-19 vaccine. The greatest difference in the intention was seen in the aged care setting, where perceived convenience was associated with a 44% (95% CI 32 to 57%) increase in the intention to vaccinate (Table 3). Participants’ work setting was the preferred location to get a COVID-19 vaccine; 86% (1550/1881) of hospital-based workers preferred vaccination in hospital, 64% (576/898) of primary care participants at a general practice, and 58% (211/365) of aged care participants preferred residential aged/disability care settings.

#### 3.3.3. Employer Requirements

Forty two percent of those working in aged care settings (42%, 153/362) felt that COVID-19 vaccination should be mandated for all healthcare workers, compared to 54% (484/893) of HCWs in primary care settings and 50% (907/1806) of HCWs in hospitals. More than half of all HCWs (57%, 1754/3058) said they would be more likely to get vaccinated if required by their employer. Of those who did not intend to get the vaccine, 23% (159/676) indicated that they would be more likely to get vaccinated if their employer required it. This potentially influenceable group included more women, more working in aged care settings and more with no comorbidities (Table S4), compared to participants who either planned to get vaccinated or did not plan to get vaccinated, and would not be influenced by an employer requirement.

#### 3.3.4. Communication Preferences

HCWs from all settings preferred to receive information from government websites or sources (62%, 1899/3074) and through discussions with their primary healthcare provider (29%, 882/3074) (Table S3). The top three most trusted spokespeople to provide information about COVID-19 vaccines for HCWs across all settings were medical professionals (73%, 2249/3074), scientists or researchers (60%, 1839/3074), and participants’ personal healthcare provider (23%, 705/3074). However, compared to hospital and primary care HCWs, those working in aged care settings reported comparatively lower levels of trust for medical professionals (61%, 224/365) and scientists (48%, 174/365).

Hospital and aged care HCWs preferred to hear about the time and location of their personal COVID-19 vaccination appointment from their employer. However, people working in primary care settings preferred their primary healthcare provider to communicate this information. Primary care providers also rated “my union or professional body” highly as a preferred information source, unlike hospital or aged care HCWs. Government representatives were not widely preferred as information sources about appointment time and location of vaccination.

Across all settings, printed materials for patients were most frequently selected to support discussions with patients (hospital 76%, 1377/1811; primary care 78%, 699/898; aged care 67%, 243/365). Approximately 61% (1878/3074) of HCWs across all settings wanted training modules providing strategies for difficult discussions. In aged care settings, online information for patients was less valued (32%, 118/365) than it was in hospital (55%, 988/1811) or primary care settings (56%, 498/989).

## 4. Discussion

This study was among the first to assess the vaccine intention and the factors driving uptake for front-line healthcare workers in Victoria, Australia at the start of the COVID-19 vaccine rollout. Our findings indicate that most (80% overall) HCWs were planning to accept a COVID-19 vaccine, but highlighted some key differences in concerns, beliefs, perceptions and communication preferences between HCWs in hospitals, primary care and aged or disability care settings.

While the majority of fatalities during Melbourne’s second wave in 2020 were in aged care settings [27], a surprisingly high proportion of HCWs in aged care did not report high concerns about passing COVID-19 to others. People working in aged or disability care settings had a lower intention to be vaccinated and were less likely to believe the vaccines were safe, important and effective than those working in hospitals or primary care settings. Other countries have also identified low rates of vaccine acceptance or uptake for non-hospital settings and patient care assistants [28]. A United States study in early 2021 found that approximately one-third of those working in nursing homes or assisted care facilities were not intending to receive (24%) or yet to decide (11%) about the COVID-19 vaccine [28]. Our participants in the aged or disability care setting also felt the least informed about key vaccine topics. Some staff in aged care settings may not have a background in health or high levels of health literacy, and so we cannot assume that they have sufficient understanding or confidence in their knowledge about vaccination [29]. Another reason for this gap may be due to the lack of appropriately tailored and translated communication materials, given that more than a quarter (27%) of our participants from aged or disability care settings spoke a language other than English at home. Inappropriate or insufficient information in different languages and for different levels of health [30] and e-Health literacy [31] has been an issue highlighted repeatedly throughout the pandemic but particularly during the vaccine rollout [32,33]. Aged and disability care workers are also more likely to be employed part time, compared with hospital or primary care HCWs, and there are fewer professional unions or organisations with wide membership across this workforce. These factors likely impact dissemination of key public health information to these workers. A key lesson for governments to take away from the COVID-19 pandemic should be the importance of establishing reliable communication channels with those working in the aged and disability care setting. This includes utilising trusted spokespeople as well as being more aware of the challenges posed by different levels of health literacy.

Half of HCWs (50%) were supportive of a vaccine requirement for all HCWs, and nearly a quarter of those who did not plan to get the vaccine said they would if their employer required it. Vaccine mandates are a potentially powerful tool to increase vaccine uptake, but key criteria should be met before implementation [34]. In particular, safe and effective vaccines must be easily accessible, culturally appropriate information and education should be provided, and less restrictive measures should be tried first [34]. While many participants supported the idea of vaccine mandates at the time of our study, other studies overseas [8,35,36] have identified resistance to these policies among healthcare workers. Results from the USA reported that only 35% of healthcare workers thought the vaccine should be mandated by their employers or the government [8]. Australia has not seen widespread resignations [37,38] in the face of recent mandates for aged care workers [39] and healthcare workers in Victoria [15] and New South Wales [16]. However, rapidly implemented vaccine mandates could damage trust [40] in government policy, which may have implications for acceptance of COVID-19 booster doses, or even uptake of other vaccines.

Our findings highlight the importance of ensuring that vaccination is convenient and accessible, particularly for prioritised workforces such as HCWs. Across all settings, participants who thought it would be convenient were significantly more likely to accept a COVID-19 vaccine. In the aged care rollout, issues arose when workers were told they could not access the vaccine on site [41,42]. Low uptake due to access barriers is often misconstrued as vaccine hesitancy. In future vaccine rollouts, it is key to assess both access and acceptance [43] and proactively develop strategies to address both.

Healthcare workers across all settings lacked information about key COVID-19 vaccine topics even as the vaccine rollout was underway. To support their discussions with patients, they wanted printed materials in preference to online information. However, the information provided needs to be easy to read, with recent reports indicating government information about vaccines is not only difficult to read [32,33], but that Australian materials are more difficult than those in the USA and UK [33]. Even HCWs in roles or settings that were not directly involved in delivering COVID-19 vaccines were willing to discuss and recommend vaccines to their patients, and they were keen for communication training to support difficult vaccine discussions. This training should reflect evidence-based recommendations to build confidence in the vaccines and communicate about vaccine risks and benefits [44,45].

### Strengths, Limitations and Future Research

Our study has a number of strengths, including being the first Australian multi-disciplinary, large-scale survey targeting healthcare workers prioritised to receive the vaccines. Our comparison of findings across three key healthcare settings is a particular strength and relatively uncommon in the global literature. This disaggregation is critical to inform targeted strategies to improve vaccine acceptance and uptake, and it will be relevant for booster dose campaigns as well as future pandemic vaccination strategies. HCWs in a range of roles and from both metropolitan and regional areas were included. During our survey period, there were no age limitations for use of specific brands of vaccine, and no major safety signals had yet emerged. Subsequently, the Australian Technical Advisory Group on Immunisation (ATAGI) recommended Pfizer as the preferred vaccine for all adults under 50 years from April 2021 [46]. Despite some media debate at the time around the effectiveness of different vaccine brands, brand preferences were not associated with intentions in the current study. However, this issue would go on to become a factor affecting uptake later in the rollout. Further limitations must also be considered. Our sample was not nationally representative, nor were our data weighted for analysis, due to the difficulties in accessing robust HCW numbers stratified by age, sex and profession. The majority of our participants were nurses, with small numbers for personal support workers and pharmacists, despite intensive efforts to recruit from these groups. This study was conducted during the first quarter of 2021 under time pressure to inform the pandemic response and at the beginning of the vaccination program rollout. We did not examine the effectiveness of communication materials in addressing HCWs’ concerns about COVID-19 vaccines, which should be the focus of future research. Consideration of utilising health professionals or pharmacists to be COVID-19 vaccine champions may facilitate uptake in lower intention or uptake groups across HCW settings.

## 5. Conclusions

Although the majority of HCWs expressed a high intention to vaccinate early in the COVID-19 vaccine rollout in Victoria, this study identified clear areas for targeted interventions across the different healthcare settings to support equitable uptake of COVID-19 and other vaccines for HCWs. Strategies such as easily accessible vaccination stations in HCWs’ workplace settings, easy-to-read and culturally appropriate information to address negative beliefs, support for healthcare workers themselves and for discussions with hesitant patients would be helpful. While generally supported, mandatory vaccination policies should be implemented with careful planning and consultation to avoid unintended consequences. By meeting vaccination uptake targets, we can be confident that our HCWs are safe and available to deliver patient care.

## Figures and Tables

**Table 1:** Demographic characteristics of participants, by setting.

Demographic Characteristics	Hospital % (*n*/N)	Primary Care % (*n*/N)	Aged Care % (*n*/N)	Total
	N = 1811	N = 898	N = 365	N = 3074
Gender ^#^				
Female	88.6 (1552/1751)	77.1 (671/870)	89.8 (309/344)	85.4 (2532/2965)
Age ^#^				
18–49	59.5 (1044/1754)	50.2 (436/869)	47.8 (163/341)	55.4 (1643/2964)
50≤	40.5 (710/1754)	49.8 (433/869)	52.2 (178/341)	44.6 (1321/2964)
Country of birth				
Born in Australia	70.2 (1272/1811)	68.5 (615/898)	57.5 (210/365)	68.2 (2097/3074)
Indigenous Australian (Aboriginal or Torres Strait Islander) ^#^				
Yes	0.5 (8/1749)	1.0 (9/860)	2.3 (8/341)	0.8 (25/2950)
Language other than English spoken at home ^#^				
Yes	13.7 (235/1717)	15.9 (134/843)	27.2 (89/327)	15.9 (458/2887)
Culturally and linguistically diverse ^^^,#^				
Yes	33.3 (592/1780)	36.9 (324/878)	47.5 (168/354)	36.0 (1084/3012)
Occupation				
Nurse	75.2 (1362/1811)	46.8 (420/898)	79.2 (289/365)	67.4 (2071/3074)
Medical Doctor	5.6 (102/1811)	7.6 (68/898)	0.3 (1/365)	5.6 (171/3074)
Pharmacist	1.2 (22/1811)	3.0 (27/898)	1.1 (4/365)	1.7 (53/3074)
Allied Health Professional	9.5 (172/1811)	6.3 (57/898)	0.8 (3/365)	7.5 (232/3074)
Personal support staff	0.2 (4/1811)	1.2 (11/898)	14.0 (51/365)	2.1 (66/3074)
Ambulance staff	0.4 (8/1811)	12.9 (116/898)	0.0 (0/365)	4.0 (124/3074)
Other	7.8 (141/1811)	22.2 (199/898)	4.7 (17/365)	11.6 (357/3074)
Employment status ^#^				
Full-time	39.2 (681/1737)	48.0 (415/864)	32.2 (109/339)	41.0 (1205/2940)
Regionality #				
Regional	20.4 (328/1609)	27.6 (221/802)	42.1 (135/321)	25.0 (684/2732)
Metropolitan	79.6 (1281/1609)	72.4 (581/802)	57.9 (186/321)	75.0 (2048/2732)
Any comorbidity ^#^				
Yes	32.4 (515/1589)	33.5 (269/803)	35.5 (110/310)	33.1 (894/2702)
Has been tested for COVID ^#^				
Yes	83.0 (1494/1800)	68.0 (609/895)	84.9 (309/364)	78.8 (2412/3059)

Note: ^#^ indicates missing data; ^^^^ CALD = born outside Australia and/or speaks a language other than English at home.

**Table 2:** Demographic characteristics associated with COVID-19 vaccine acceptance, by total sample.

Characteristic	All HCWs, N	Would Get the COVID-19 Vaccine? % (*n*)			Unadjusted Risk Difference (Difference in Proportion) ^^,^*	95% CI	*p*-Value
		Yes	Not Sure	No			
Total	3074	77.9 (2394)	14.0 (429)	8.1 (251)	N/A		
Gender ^#^							
Female	2532	78.3 (1982)	14.4 (365)	7.3 (185)			
Male	392	85.5 (335)	7.7 (30)	6.9 (27)	7.2	3.3 to 11.0	**<0.001**
Prefer not to say	41	31.7 (13)	24.4 (10)	43.9 (18)	−46.6	−60.9 to −32.2	**<0.001**
Age ^#^							
18–49	1643	74.1 (1217)	16.3 (268)	9.6 (158)			
50=<	1321	84.4 (1115)	10.4 (138)	5.1 (68)	10.3	7.5 to 13.2	**<0.001**
Country of birth							
Born in Australia	2097	78.4 (1645)	13.3 (279)	8.2 (173)			
Not born in Australia	977	76.7 (749)	15.4 (150)	8.0 (78)	−1.8	−5.0 to 1.4	0.272
Aboriginal or Torres Strait Islander ^#^							
Yes	25	60.0 (15)	24.0 (6)	16.0 (4)			
No	2893	79.4 (2297)	13.4 (387)	7.2 (209)	19.4	0.1 to 38.7	**0.048**
Prefer not to say	32	34.4 (11)	28.1 (9)	37.5 (12)	−25.6	−50.9 to −0.3	**0.047**
Language other than English spoken at home ^#^							
Yes	458	75.5 (346)	17.2 (79)	7.2 (33)			
No	2429	79.1 (1921)	13.1 (318)	7.8 (190)	3.5	−0.7 to 7.8	0.103
Culturally and linguistically diverse ^^^,#^							
Yes	1084	76.7 (831)	15.1 (164)	8.2 (89)			
No	1928	78.4 (1511)	13.4 (258)	8.2 (159)	1.7	−1.4 to 4.8	0.282
Occupation							
Nurse	2071	76.9 (1592)	14.4 (299)	8.7 (180)			
Medical Doctor	171	93.6 (160)	4.1 (7)	2.3 (4)	16.7	12.6 to 20.8	**<0.001**
Pharmacist	53	96.2 (51)	1.9 (1)	1.9 (1)	19.4	13.9 to 24.8	**<0.001**
Allied Health Professional	232	76.3 (177)	17.2 (40)	6.5 (15)	−0.6	−6.3 to 5.2	0.844
Personal support staff	66	56.1 (37)	25.8 (17)	18.2 (12)	−20.8	−32.9 to −8.7	**0.001**
Ambulance staff	124	79.8 (99)	12.9 (16)	7.3 (9)	3.0	−4.3 to 10.3	0.425
Other	357	77.9 (278)	13.7 (49)	8.4 (30)	1.0	−3.7 to 5.7	0.675
Occupation settings ^#^							
Hospital	1811	77.1 (1396)	14.4 (260)	8.6 (155)			
Primary care (community or private practice)	898	84.1 (755)	10.7 (96)	5.2 (47)	7.0	3.9 to 10.1	**<0.001**
Residential aged or disability care facility	365	66.6 (243)	20.0 (73)	13.4 (49)	−10.5	−15.7 to −5.3	**<0.001**
Employment status ^#^							
Full-time	1205	81.4 (981)	11.8 (142)	6.8 (82)			
Part-time	1735	76.9 (1334)	15.0 (261)	8.1 (140)	−4.5	−7.5 to −1.6	**0.003**
Regionality ^#^							
Regional	684	76.3 (522)	15.1 (103)	8.6 (59)			
Metropolitan	2048	82.0 (1680)	12.5 (256)	5.5 (112)	5.7	2.1 to 9.3	**0.002**
Any comorbidity ^#^							
Yes	894	80.4 (719)	13.4 (120)	6.2 (55)			
No	1808	78.5 (1420)	13.8 (250)	7.6 (138)	−1.9	−5.1 to 1.3	0.251
Has been tested for COVID ^#^							
Yes	2412	79.7 (1922)	14.1 (339)	6.3 (151)			
No	647	71.3 (461)	13.4 (87)	15.3 (99)	−8.4	−12.3 to −4.6	**<0.001**

Note: Bold typeface indicates statistical significance where *p* < 0.05. ^^^ Risk difference comparisons are Yes vs. Not sure/No, * Comparison between intention to accept COVID-19 vaccine was estimated using binary regression. ^^^^ Culturally and linguistically diverse = born outside Australia and/or speaks a language other than English at home; ^#^ indicates missing data.

**Table 3:** Thoughts and feelings and practical issues related to COVID-19 vaccination and associations with intentions, by HCW setting.

Thoughts and Feelings and Practical Issues	Would Get the COVID-19 Vaccine?Yes, % (*n*/N)			Adjusted Risk Difference ^^,^* (95% CI, *p*-Value)		
	Hospital	Primary Care	Aged Care	Hospital	Primary Care	Aged Care
	N = 1811	N = 898	N = 365			
Perceived risks of COVID-19						
Concerned about getting COVID-19 ^^^^						
Not at all/A little	75.1 (789/1050)	82.4 (430/522)	69.5 (146/210)			
Very/Moderately	79.9 (605/757)	86.7 (325/375)	62.6 (97/155)	2.9 (−0.9 to 6.6; 0.139)	4.2 (0.1 to 8.4; 0.050)	4.2 (−14.8 to 6.3; 0.430)
Concerned about your patients or residents getting COVID-19 from you ^^^^						
Not at all/A little	71.5 (656/917)	81.5 (358/439)	60.1 (89/148)			
Very/Moderately	82.9 (739/891)	86.4 (394/456)	71.6 (154/215)	8.6 (4.9 to 12.3; <0.001)	2.4 (−1.9 to 6.7; 0.260)	−1.4 (−11.6 to 8.8; 0.790)
Beliefs about the COVID-19 vaccine						
Trust the new COVID-19 vaccines ^^^^^						
Not at all/A little	23.6 (101/428)	25.5 (35/137)	15.7 (20/127)			
Very/moderately	93.6 (1290/1378)	94.6 (717/758)	93.7 (222/237)	66.3(61.3 to 71.2; <0.001)	63.2 (54.0 to 72.5; <0.001)	50.7 (50.7 to 50.7; <0.001)
Think getting a COVID-19 vaccine will be important for your health ^^^^^						
Not at all/A little	25.4 (88/346)	25.7 (35/136)	16.1 (18/112)			
Very/moderately	89.4 (1296/1450)	94.6 (718/759)	89.2 (223/250)	60.5 (54.8 to 66.2; <0.001)	59.5 (49.9 to 69.0; <0.001)	36.7 (−92.4 to 165.9; 0.580)
Think getting a COVID-19 vaccine will protect other people in your community from COVID-19 ^^^^^						
Not at all/A little	21.6 (63/292)	28.2 (33/117)	12.0 (12/100)			
Very/moderately	87.8 (1321/1504)	92.5 (712/770)	87.4 (229/262)	61.5 (55.4 to 67.7; <0.001)	41.1 (31.2 to 51.0; <0.001)	58.8 (58.7 to 58.8; <0.001)
Think a COVID-19 vaccine will be safe for you ^^^^^						
Not at all/A little	23.9 (104/436)	27.8 (40/144)	12.9 (15/116)			
Very/moderately	94.0 (1279/1361)	95.0 (709/746)	91.9 (226/246)	67.0 (62.0 to 71.9; <0.001)	59.6 (50.3 to 68.9; <0.001)	52.1 (47.4 to 56.8; <0.001)
Concerned that a COVID-19 vaccine could cause you to have a serious reaction ^^^^^						
Not at all/A little	51.5 (333/647)	62.4 (156/250)	47.6 (91/191)			
Very/moderately	91.4 (1055/1154)	92.5 (594/642)	87.8 (151/172)	37.6 (−42.1 to −33.1; <0.001)	22.1 (15.4 to 28.7; <0.001)	37.6 (28.1 to 47.1; <0.001)
Sufficient information about COVID-19 vaccines						
Not informed about at least one of the five issues	71.2 (945/1328)	79.5 (495/623)	58.5 (155/265)			
Informed about all five issues	94.8 (438/462)	95.5 (257/269)	88.8 (87/98)	19.9 (17.4 to 22.4; <0.001)	13.4 (9.4 to 17.4; <0.001)	32.2 (25.0 to 39.4; <0.001)
Perceived convenience of getting a COVID-19 vaccine						
Not at all/A little	48.8 (137/281)	71.5 (133/186)	29.1 (25/86)			
Very/moderately	82.3 (1250/1519)	87.4 (619/708)	79.0 (218/276)	29.6 (22.7 to 36.5; <0.001)	11.1 (4.4 to 17.9; <0.001)	44.4 (32.2 to 56.6; <0.001)

Note: ^^^ Risk difference comparisons are Yes vs. Not sure/No, * Comparison between intention to accept COVID-19 vaccine was estimated using binary regression adjusting for age, sex, culturally and linguistically diverse, employment status and regionality. ^^^^ Perceived risks of COVID-19. ^^^^^ Beliefs about the COVID-19 vaccine.

## Data Availability

Restrictions apply to the availability of these data. Data are available from the authors upon request and with the permission of the Victorian Department of Health.

## Supplementary Materials

The following are available online at https://www.mdpi.com/article/10.3390/vaccines10010003/s1: Table S1: Survey items and sources; Table S2: Factors influencing the decision, by total sample; Table S3: All descriptive analyses; Table S4: Demographic characteristics by intention to vaccinate and/or required to vaccinate by employer.
