# Supplementary material for: Factors Influencing Australian Healthcare Workers’ COVID-19 Vaccine Intentions across Settings: A Cross-Sectional Survey
Source: Vaccines (Basel). 2021 Dec 21;10(1):3. doi: 10.3390/vaccines10010003 (PMC8781521; doi:10.3390/vaccines10010003)
Supplement: Supplementary file 1 [file vaccines-10-00003-s001.zip › vaccines-1505533-supplementary.pdf]

SUPPLEMENTAL MATERIAL

Table S1: Survey items and sources

Category	Question	Response / scoring	Source
Occupation	Are you a healthcare worker currently working in Victoria, Australia?	• Yes<br>• No	
Occupation	What is your primary professional role?	• Nurse (e.g. nurse educator, midwife, registered nurse, enrolled nurse)<br>• Medical doctor (e.g. General Practitioner, non-GP medical specialist, visiting medical officer, intern, hospital medical officer, GP registrar)<br>• Pharmacist<br>• Allied health professional (e.g. physiotherapist, occupational therapist)<br>• Personal service worker (e.g. dental hygienist, Aboriginal and/or Torres Strait Islander health worker, massage therapist, nursing support, personal care worker)<br>• Ambulance staff or paramedic<br>• Other health professional (e.g. dentist, psychologist, radiographer, medical scientist, medical technician, radiation therapist)<br>• Other (please specify) [open text]	
Occupation	In which setting do you predominantly work?	• Hospital<br>• Community<br>• Residential aged or disability care facility<br>• Other (please specify) [open text]	
COVID-19 testing	Have you ever been tested for COVID-19?	• No<br>• Yes	
Perceived risk of COVID-19	How concerned are you about getting COVID-19?	• Not at all concerned<br>• A little concerned<br>• Moderately concerned<br>• Very concerned	BeSD

Perceived risk of COVID-19	How concerned are you about your patients or residents getting COVID-19 from you?	• Not at all concerned<br>• A little concerned<br>• Moderately concerned<br>• Very concerned	BeSD
Vaccine beliefs	How much do you trust the new COVID-19 vaccines?	• Not at all<br>• A little<br>• Moderately<br>• Very much	BeSD
Vaccine beliefs	How important do you think getting a COVID-19 vaccine will be for your health?	• Not at all important<br>• A little important<br>• Moderately important<br>• Very important	BeSD
Vaccine beliefs	How much do you think getting a COVID-19 vaccine for yourself will protect other people in your community from COVID-19?	• Not at all<br>• A little<br>• Moderately<br>• Very much	BeSD
Vaccine beliefs	How safe do you think a COVID-19 vaccine will be for you?	• Not at all safe<br>• A little safe<br>• Moderately safe<br>• Very safe	BeSD
Vaccine beliefs	How concerned are you that a COVID-19 vaccine could cause you to have a serious reaction? Would you say…	• Not at all concerned<br>• A little concerned<br>• Moderately concerned<br>• Very concerned	BeSD
Vaccine intention	If a COVID-19 vaccine were recommended for you, would you get it?	• No [Sub-question A.1]<br>• Yes<br>• Not sure [Sub-question A.1]	BeSD
Vaccine concerns	[Sub-question A.1]<br>If no or unsure, what are your reasons for your decision (tick all that apply)	• I don't believe I'm at risk<br>• I am concerned about minor side effects<br>• I am concerned about serious reactions<br>• I am concerned that the vaccines haven't been tested enough for safety	

		• I am concerned about the potential long-term effects of the vaccine<br>• I am concerned about safety in pregnancy<br>• I am concerned about safety whilst breastfeeding<br>• I am concerned about pain from the needle<br>• I have had a reaction to a vaccine in the past<br>• I am concerned about needing to take time off work<br>• I am concerned that the vaccine won't work well enough<br>• Other [open text]	
Perceived convenience	How convenient do you think it will be for you to get a COVID-19 vaccine?	• Not at all convenient [Sub-question B.1]<br>• A little convenient [Sub-question B.1]<br>• Moderately convenient [Sub-question B.1]<br>• Very convenient	Adapted from BeSD
Perceived convenience	[Sub-question B.1]<br>If not at all/a little/moderately convenient, what do you think will make it hard for you to get a COVID-19 vaccine? (tick all that apply)	• Knowing which vaccine priority group I am in (e.g. Phase 1a, Phase 1b etc)<br>• Knowing where to go to get the vaccine<br>• Organising a vaccine appointment at a time that suits me<br>• Travelling to a location where I can get a vaccine<br>• Waiting a long time at the location<br>• Taking time off work<br>• Managing carer or family responsibilities (childcare etc)<br>• Something else, please specify [open text]	
Vaccine requirements	Do you think COVID-19 vaccines should be mandated for healthcare workers?	• No<br>• Yes, for all healthcare workers<br>• Yes, for healthcare workers in high-risk settings only (e.g. ICU, ED, residential aged care)<br>• Not sure	
Vaccine requirements	If your employer requires you to get a COVID-19 vaccine, will this make you more likely to get it?	• No<br>• Yes<br>• Not sure	
Preferred location	Where would you prefer to get a COVID-19 vaccine?	• Hospital<br>• General practice<br>• Pharmacy	Adapted from BeSD

		• Community centre, meeting hall, or local shop<br>• Large public space (e.g. conference centre, stadium)<br>• Council clinic<br>• Place of worship<br>• Residential aged care or disability care facility<br>• Somewhere else, please specify [open text]<br>• n/a	
Factors influencing the decision	Which of the following factors might influence your decision about getting the COVID-19 vaccine? (tick all that apply)	• Brand of vaccine offered [Sub-questions C.1, C.2]<br>• Country where the vaccine was manufactured [Sub-question C.3]<br>• Information about the vaccine approval process<br>• The reported efficacy of the vaccine from clinical trials<br>• The reported safety of the vaccine from clinical trials<br>• Seeing how people who have been vaccinated overseas have reacted to the vaccine<br>• If the vaccine is recommended by my professional society (e.g. Royal Australian College of General Practitioners, Australian Primary Health Care Nurse Association, Pharmaceutical Society of Australia)<br>• If the vaccine is available at my workplace<br>• If the vaccine is required to travel overseas<br>• Other [open text]	
Factors influencing the decision	[Sub-question C.1]<br>If yes, which brand would you prefer?	• Pfizer<br>• Oxford/AstraZeneca<br>• Novavax<br>• Other COVID-19 vaccines purchased through COVAX facility, such as Moderna	
Perceived factors influencing the decision	[Sub-question C.2]<br>If you can't get your brand of choice, would you be willing to get another brand?	• Yes<br>• No<br>• Not sure	
Perceived factors influencing the decision	[Sub-question C.3]<br>If yes, which country would you prefer?	• Made in Australia<br>• Made in the USA<br>• Made in Europe/UK<br>• Made in Russia	

		• Made in another country [open text]	
Information about COVID-19 vaccines	For each of the following topics on COVID-19 vaccines, do you feel you have enough information?		
Information about COVID-19 vaccines	How the COVID vaccines work	• Yes<br>• No<br>• Unsure	
Information about COVID-19 vaccines	How effective the COVID vaccines are	• Yes<br>• No<br>• Unsure	
Information about COVID-19 vaccines	How safe the COVID vaccines are	• Yes<br>• No<br>• Unsure	
Information about COVID-19 vaccines	The vaccine side effects	• Yes<br>• No<br>• Unsure	
Information about COVID-19 vaccines	The COVID vaccine recommendations (e.g. number of doses, spacing)	• Yes<br>• No<br>• Unsure	
Communication preferences	How do you prefer to receive COVID-19 vaccine information? (tick your top two)	• Television [Sub-question D.1]<br>• Radio [Sub-question D.2]<br>• Government website or sources<br>• Other website [open text]<br>• Newspapers (online and print)<br>• Academic journals<br>• Printed materials<br>• Social media [Sub-question D.3]<br>• Hotline to ring for information<br>• Discussion with my healthcare provider	

		• Family or friends<br>• Other [open text]	
Communication preferences	[Sub-question D.1]<br>Television	• Community/public<br>• Commercial	
Communication preferences	[Sub-question D.2]<br>Radio	• Community/public<br>• Commercial	
Communication preferences	[Sub-question D.3]<br>Social media	• Facebook<br>• Twitter<br>• WhatsApp<br>• WeChat<br>• Other [open text]	
Communication preferences	Who do you trust most to provide you with information about the COVID-19 vaccine? (tick your top two)	• Scientists or researchers<br>• Medical professionals<br>• My primary healthcare provider<br>• Commonwealth Government representative<br>• State Government representative<br>• Celebrities or online influencers<br>• Community leaders<br>• Religious leaders<br>• Family or friends<br>• Other [open text]	
Communication preferences	Who would you prefer to inform you about the timing and location of your vaccination? (tick your top two)	• Commonwealth Government representative<br>• State Government representative<br>• My local council<br>• My primary healthcare provider<br>• My employer<br>• My union or professional body<br>• Community health worker<br>• Local hospital infectious disease or immunisation department	
Communication preferences	Please select the resources you think would be most helpful to support you	• Printed information for patients<br>• Online information for patients	

	discussing, recommending and/or delivering COVID-19 vaccines to eligible patients? (tick all that apply)	• Training modules providing strategies for difficult discussions about COVID-19 vaccines e.g. guidance on speaking with a vaccine hesitant patient<br>• Resources to support informed consent<br>• Telephone hotline offering COVID-19 vaccine information for healthcare providers<br>• Other [open text]	
Vaccine recommendation	Would you recommend COVID-19 vaccination to eligible patients or residents?	• No<br>• Yes<br>• Not sure [Sub-question E.1]	BeSD
	[Sub-question E.1]<br>If no or unsure, why not?	Open text	
Demographics	What is your age group?	• 18 - 29<br>• 30 - 39<br>• 40 – 49<br>• 50 - 59<br>• 60 - 69<br>• 70 – 79<br>• 80+	
Demographics	What is your gender?	• Woman<br>• Man<br>• Non-binary or gender fluid<br>• Prefer not to say	
Demographics	What is your country of birth?	Drop-down menu of countries	
Demographics	Do you speak a language other than English at home most of the time?	• No<br>• Yes [Sub-question F.1]	
Demographics	[Sub-question F.1]<br>If yes, what language?	Open text	
Demographics	Are you of Aboriginal or Torres Strait Islander origin?	• No<br>• Yes<br>• Prefer not to say	

Demographics	What is the postcode where you live?	Open text	
Employment	Which of the following best describes your current employment?	• Full-time [Sub-question G.1]<br>• Part-time [Sub-question G.1]<br>• Other (please specify) [open text]	
Employment	[Sub-question G.1]<br>Full-time OR part-time	• Casual<br>• Fixed term contract<br>• Continuing position<br>• Self-employed/contractor	
Employment	How many years of experience do you have in your current profession?	Open text	
Comorbidities	Do you currently have any of the following conditions (tick all that apply)	• Cardiovascular disease (e.g. Heart disease, history of heart attack or stroke)<br>• Respiratory disease or chronic respiratory condition (e.g. severe asthma, COPD, emphysema, other lung disease)<br>• Chronic neurological conditions (e.g. multiple sclerosis, spinal cord injuries, seizure disorders)<br>• Cancer or history of cancer<br>• Diabetes (type 1 or type 2)<br>• Autoimmune disease (e.g. lupus, multiple sclerosis, rheumatoid arthritis, psoriasis, Crohn's disease, inflammatory bowel disease)<br>• Immunocompromising condition (e.g. HIV, cancer, transplantation, regular steroid use)<br>• Chronic kidney disease<br>• Chronic liver disease<br>• Other, please specify [open text]<br>• Prefer not to say<br>• None of the above	

Table S2: Factors influencing the decision, by total sample

	Would get the COVID-19 vaccine? Yes, % (n)	Risk difference[#]	95% CI	p-value
The reported safety of the vaccine from clinical trials				
No	71.0 (590/831)			
Yes	80.4 (1804/2243)	6.1	2.7 to 9.6	<0.001
The reported efficacy of the vaccine from clinical trials				
No	68.5 (619/904)			
Yes	81.8 (1775/2170)	9.7	6.3 to 13.2	<0.001
Seeing how people overseas have reacted to the vaccine				
No	79.0 (1079/1366)			
Yes	77.0 (1315/1708)	-2.6	-5.5 to 0.2	0.066
If the vaccine is recommended by my professional society				
No	66.7 (1024/1536)			
Yes	89.1 (1370/1538)	17.5	14.7 to 20.4	<0.001
If the vaccine is available at my workplace				
No	65.3 (1081/1655)			
Yes	92.5 (1313/1419)	20.9	18.3 to 23.5	<0.001
Information about the vaccine approval process				
No	77.0 (1309/1700)			
Yes	79.0 (1085/1374)	1.1	-1.6 to 4.0	0.422
If the vaccine is required to travel overseas				
No	74.0 (1370/1852)			
Yes	83.8 (1024/1222)	7.5	4.9 to 10.1	<0.001
Brand of vaccine offered				
No	77.7 (1681/2164)			
Yes	78.4 (713/910)	-2.0	-5.1 to 1.1	0.206
Country where the vaccine was manufactured				
No	78.7 (1896/2410)			
Yes	75.0 (498/664)	-3.2	-6.8 to 0.3	0.075

Comparison between intention to accept COVID-19 vaccine was estimated using binary regression adjusting for age, sex, Culturally and linguistically diverse, employment status and regionality.

Table S3: All descriptive analyses

	Hospital	Primary	Aged Care	Total
	N=1811	N=898	N=365	N=3074
	**% (n/N)**	**% (n/N)**	**% (n/N)**	**% (n/N)**
Would you recommend COVID-19 vaccination to eligible patients				
Yes	80.3 (1412/1759)	85.7 (749/874)	65.2 (227/348)	80.1 (2388/2981)
Perceived influences on COVID vaccine decision				
The reported safety of the vaccine from clinical trials	73.7 (1334/1811)	74.9 (673/898)	64.7 (236/365)	73.0 (2243/3074)
The reported efficacy of the vaccine from clinical trials	71.1 (1287/1811)	74.3 (667/898)	59.2 (216/365)	70.6 (2170/3074)
Seeing how people overseas have reacted to the vaccine	56.3 (1019/1811)	56.8 (510/898)	49.0 (179/365)	55.6 (1708/3074)
If the vaccine is recommended by my professional society	49.6 (898/1811)	53.9 (484/898)	42.7 (156/365)	50.0 (1538/3074)
If the vaccine is available at my workplace	50.6 (917/1811)	40.8 (366/898)	37.3 (136/365)	46.2 (1419/3074)
Information about the vaccine approval process	46.1 (834/1811)	43.7 (392/898)	40.5 (148/365)	44.7 (1374/3074)
If the vaccine is required to travel overseas	41.1 (745/1811)	40.2 (361/898)	31.8 (116/365)	39.8 (1222/3074)
Brand of vaccine offered	29.5 (535/1811)	30.2 (271/898)	28.5 (104/365)	29.6 (910/3074)
Country where the vaccine was manufactured	20.5 (372/1811)	23.1 (207/898)	23.3 (85/365)	21.6 (664/3074)
Concerns for those not intending to get a COVID vaccine	**N=415**	**N=143**	**N=122**	**N=699**
I do not believe I am at risk	10.6 (44/415)	18.9 (27/143)	11.5 (14/122)	12.5 (85/680)
I am concerned about minor side effects	19.8 (82/415)	18.2 (26/143)	23.0 (28/122)	20.0 (136/680)
I am concerned about serious reactions	50.8 (211/415)	47.6 (68/143)	52.5 (64/122)	50.4 (343/680)
I am concerned that the vaccines haven't been tested enough	73.5 (305/415)	67.1 (96/143)	78.7 (96/122)	73.1 (497/680)
I am concerned about the potential long-term effects of the	68.4 (284/415)	35.7 (51/143)	72.1 (88/122)	62.2 (423/680)
I am concerned about safety in pregnancy	28.9 (120/415)	25.2 (36/143)	13.9 (17/122)	25.4 (173/680)
I am concerned about safety whilst breastfeeding	12.5 (52/415)	13.3 (19/143)	4.9 (6/122)	11.3 (77/680)
I am concerned about pain from the needle	0.7 (3/415)	0.7 (1/143)	0.0 (0/122)	0.6 (4/680)
I have had a reaction to a vaccine in the past	12.0 (50/415)	8.4 (12/143)	12.3 (15/122)	11.3 (77/680)
I am concerned about needing to take time off work	8.7 (36/415)	6.3 (9/143)	13.9 (17/122)	9.1 (62/680)
I am concerned that the vaccine won't work well enough	29.2 (121/415)	37.1 (53/143)	36.1 (44/122)	32.1 (218/680)
Other	10.1 (42/415)	13.3 (19/143)	8.2 (10/122)	10.4 (71/680)

Perceived risks of COVID-19				
Concerned about getting COVID-19				
Very / Moderately	41.9 (757/1807)	41.8 (375/897)	42.5 (155/365)	41.9 (1287/3069)
Concerned about your patients or residents getting COVID-19 from you				
Very / Moderately	49.3 (891/1808)	50.9 (456/895)	59.2 (215/363)	50.9 (1562/3066)
Beliefs				
Trust the new COVID-19 vaccines (very/moderately)				
Very/moderately	76.3 (1378/1806)	84.7 (758/895)	65.1 (237/364)	77.4 (2373/3065)
Think getting a COVID-19 vaccine will be important for your health				
Very/moderately	80.7 (1450/1796)	84.8 (759/895)	69.1 (250/362)	80.5 (2459/3053)
Think getting a COVID-19 vaccine will protect other people in your community from COVID-19				
Very/moderately	83.7 (1504/1796)	86.8 (770/887)	72.4 (262/362)	83.3 (2536/3045)
Think a COVID-19 vaccine will be safe for you				
Very/moderately	75.7 (1361/1797)	83.8 (746/890)	68.0 (246/362)	77.2 (2353/3049)
Concerned that a COVID-19 vaccine could cause you to have a serious reaction				
Very/moderately	35.9 (647/1801)	28.0 (250/892)	52.6 (191/363)	35.6 (1088/3056)
Sufficient information about …				
How the COVID vaccines work	53.3 (953/1789)	60.2 (536/891)	49.6 (180/363)	54.8 (1669/3043)
How effective the COVID vaccines are	50.7 (905/1784)	58.1 (517/890)	39.4 (143/363)	51.5 (1565/3037)
How safe the COVID vaccines are	48.7 (869/1785)	53.7 (477/888)	37.4 (135/361)	48.8 (1481/3034)
The vaccine side effects	42.5 (749/1764)	46.1 (408/885)	37.8 (136/360)	43.0 (1293/3009)
The vaccine recommendations	58.8 (1048/1783)	61.0 (539/884)	57.1 (204/357)	59.2 (1791/3024)
Totally informed (yes to all 5)	25.8 (462/1790)	30.2 (269/892)	27.0 (98/363)	27.2 (829/3045)
Perceived convenience of getting a COVID-19 vaccine				
Very/moderately	84.4 (1519/1800)	79.2 (708/894)	76.2 (276/362)	81.9 (2503/3056)
Challenges for those perceiving vaccination as less than very convenient				

Knowing which vaccine priority group I am in	32.9 (297/903)	26.4 (151/571)	21.0 (48/229)	29.1 (496/1703)
Knowing where to go to get the vaccine	21.4 (193/903)	30.8 (176/571)	21.8 (50/229)	24.6 (419/1703)
Organising a vaccine appointment at a time that suits me	44.6 (403/903)	48.5 (277/571)	34.1 (78/229)	44.5 (758/1703)
Travelling to a location where I can get a vaccine	25.6 (231/903)	32.2 (184/571)	27.5 (63/229)	28.1 (478/1703)
Waiting a long time at the location	30.6 (276/903)	40.5 (231/571)	23.6 (54/229)	32.9 (561/1703)
Taking time off work	19.7 (178/903)	25.9 (148/571)	13.5 (31/229)	21.0 (357/1703)
Managing carer or family responsibilities	13.3 (120/903)	12.4 (71/571)	14.8 (34/229)	13.2 (225/1703)
Something else	11.2 (101/903)	7.2 (41/571)	16.2 (37/229)	10.5 (179/1703)
Think COVID-19 vaccines should be mandated for all healthcare workers				
Yes for all	50.2 (907/1806)	54.2 (484/893)	42.3 (153/362)	50.4 (1544/3061)
Will be more likely to get a COVID-19 vaccine if required by employer				
Yes	55.9 (1009/1804)	57.9 (515/890)	63.2 (230/364)	57.4 (1754/3058)
Preferred place to get a COVID-19 vaccine				
Hospital	85.6 (1550/1811)	46.8 (420/898)	29.3 (107/365)	67.6 (2077/3074)
General practice	33.1 (600/1811)	64.1 (576/898)	34.2 (125/365)	42.3 (1301/3074)
Pharmacy	15.1 (274/1811)	24.5 (220/898)	9.0 (33/365)	17.1 (527/3074)
Community centre, meeting hall, or local shop	10.4 (188/1811)	21.4 (192/898)	4.9 (18/365)	12.9 (398/3074)
Large public space (e.g. conference centre, stadium)	12.0 (218/1811)	18.6 (167/898)	6.6 (24/365)	13.3 (409/3074)
Council clinic	13.7 (249/1811)	27.5 (247/898)	9.9 (36/365)	17.3 (532/3074)
Place of worship	1.7 (30/1811)	3.3 (30/898)	2.5 (9/365)	2.2 (69/3074)
Residential aged care or disability care facility	3.0 (55/1811)	3.5 (31/898)	57.8 (211/365)	9.7 (297/3074)
Somewhere else	2.8 (51/1811)	5.5 (49/898)	0.8 (3/365)	3.4 (103/3074)
Preferred platform to receive information				
Television	8.9 (162/1811)	9.0 (81/898)	11.8 (43/365)	9.3 (286/3074)
Radio	3.1 (56/1811)	4.9 (44/898)	2.5 (9/365)	3.5 (109/3074)
Government website or sources	61.6 (1115/1811)	67.4 (605/898)	49.0 (179/365)	61.8 (1899/3074)
Other website	3.9 (71/1811)	5.2 (47/898)	3.0 (11/365)	4.2 (129/3074)
Newspapers (online and print)	7.3 (132/1811)	8.9 (80/898)	7.9 (29/365)	7.8 (241/3074)

Academic journals	25.4 (460/1811)	17.3 (155/898)	20.0 (73/365)	22.4 (688/3074)
Printed materials	22.5 (408/1811)	21.0 (189/898)	26.6 (97/365)	22.6 (694/3074)
Social media	7.0 (127/1811)	6.1 (55/898)	6.6 (24/365)	6.7 (206/3074)
Hotline to ring for information	3.6 (66/1811)	4.7 (42/898)	6.0 (22/365)	4.2 (130/3074)
Discussion with my health care provider	29.0 (526/1811)	27.4 (246/898)	30.1 (110/365)	28.7 (882/3074)
Family or friends	1.0 (19/1811)	1.1 (10/898)	1.1 (4/365)	1.1 (33/3074)
Other	4.5 (81/1811)	5.6 (50/898)	3.0 (11/365)	4.6 (142/3074)
Trusted source of information				
Scientists or researchers	61.8 (1119/1811)	60.9 (547/898)	47.7 (174/365)	59.9 (1840/3074)
Medical professionals	74.5 (1350/1811)	75.2 (675/898)	61.4 (224/365)	73.2 (2249/3074)
My primary healthcare provider	22.0 (399/1811)	22.9 (206/898)	27.4 (100/365)	22.9 (705/3074)
Commonwealth Government representative	8.4 (153/1811)	13.0 (117/898)	14.0 (51/365)	10.4 (321/3074)
State Government representative	8.2 (149/1811)	7.9 (71/898)	11.8 (43/365)	8.6 (263/3074)
Celebrities or online influencers	0.1 (2/1811)	0.1 (1/898)	0.3 (1/365)	0.1 (4/3074)
Community leaders	0.4 (7/1811)	0.3 (3/898)	0.5 (2/365)	0.4 (12/3074)
Religious leaders	0.2 (3/1811)	0.1 (1/898)	0.5 (2/365)	0.2 (6/3074)
Family or friends	0.8 (14/1811)	0.6 (5/898)	0.8 (3/365)	0.7 (22/3074)
Other	2.2 (39/1811)	2.3 (21/898)	3.3 (12/365)	2.3 (72/3074)
Preferred source of information for vaccination time and location				
Commonwealth Government representative	5.4 (97/1811)	10.6 (95/898)	8.5 (31/365)	7.3 (223/3074)
State Government representative	9.2 (166/1811)	17.0 (153/898)	11.5 (42/365)	11.7 (361/3074)
My local council	2.6 (47/1811)	9.1 (82/898)	3.0 (11/365)	4.6 (140/3074)
My primary healthcare provider	30.2 (547/1811)	40.8 (366/898)	36.7 (134/365)	34.1 (1047/3074)
My employer	59.7 (1082/1811)	38.6 (347/898)	51.0 (186/365)	52.5 (1615/3074)
My union or professional body	13.0 (235/1811)	24.5 (220/898)	14.5 (53/365)	16.5 (508/3074)
Community health worker	1.7 (30/1811)	4.3 (39/898)	2.5 (9/365)	2.5 (78/3074)
Local hospital infectious disease or immunisation department	44.7 (810/1811)	26.5 (238/898)	24.9 (91/365)	37.1 (1139/3074)
Helpful resources to support discussions				
Printed information for patients	76.0 (1377/1811)	77.8 (699/898)	66.6 (243/365)	75.4 (2319/3074)

Online information for patients	54.6 (988/1811)	55.5 (498/898)	31.8 (116/365)	52.1 (1602/3074)
Training modules providing strategies for difficult discussi**ons**	60.1 (1088/1811)	64.4 (578/898)	58.1 (212/365)	61.1 (1878/3074)
Resources to support informed consent	53.0 (960/1811)	53.0 (476/898)	51.0 (186/365)	52.8 (1622/3074)
Telephone hotline offering COVID-19 vaccine information for	36.6 (662/1811)	37.4 (336/898)	28.2 (103/365)	35.8 (1101/3074)
Other	2.2 (39/1811)	3.1 (28/898)	3.6 (13/365)	2.6 (80/3074)

Table S4: Demographic characteristics by intention to vaccinate and/or required to vaccinate by employer

	Not intending and would not be influenced by employer requirement	Intending to vaccinate and would be influenced by employer requirement	Intending to vaccinate but would not be influenced by employer requirement	**Not intending but would be influenced by employer requirement**
	N=517	N=1595	N=787	N=159
Gender				
**Female**	85.2 (415/487)	86.5 (1344/1553)	82.1 (629/766)	91.7 (133/145)
**Male**	9.7 (47/487)	13.2 (205/1553)	16.7 (128/766)	6.2 (9/145)
**Prefer not to say**	5.1 (25/487)	0.3 (4/1553)	1.2 (9/766)	2.1 (3/145)
Age				
**18-49**	67.3 (325/483)	51.2 (796/1554)	54.2 (416/767)	67.1 (98/146)
**50=<**	32.7 (158/483)	48.8 (758/1554)	45.8 (351/767)	32.9 (48/146)
Country of birth				
**Born in Australia**	68.1 (352/517)	65.1 (1039/1595)	76.1 (599/787)	62.3 (99/159)
**Not born in Australia**	31.9 (165/517)	34.9 (556/1595)	23.9 (188/787)	37.7 (60/159)
Indigenous Australian (Aboriginal or Torres Strait Islander)				
**Yes**	1.7 (8/480)	0.6 (10/1547)	0.7 (5/766)	1.4 (2/144)
**No**	94.8 (455/480)	99.2 (1534/1547)	98.3 (753/766)	95.8 (138/144)
**Prefer not to say**	3.5 (17/480)	0.2 (3/1547)	1.0 (8/766)	2.8 (4/144)
Only English spoken at home				
**Yes**	17.5 (83/474)	18.6 (281/1512)	8.3 (62/744)	19.6 (28/143)
**No**	82.5 (391/474)	81.4 (1231/1512)	91.7 (682/744)	80.4 (115/143)
Culturally and linguistically diverse				
**Yes**	36.9 (188/510)	38.7 (606/1566)	28.8 (220/764)	39.7 (62/156)
**No**	63.1 (322/510)	61.3 (960/1566)	71.2 (544/764)	60.3 (94/156)

Occupation				
**Nurse**	71.2 (368/517)	69.5 (1109/1595)	60.7 (478/787)	68.6 (109/159)
**Medical Doctor**	1.4 (7/517)	4.9 (78/1595)	10.2 (80/787)	2.5 (4/159)
**Pharmacist**	0.4 (2/517)	1.9 (31/1595)	2.5 (20/787)	0.0 (0/159)
**Allied Health Prof**	7.4 (38/517)	6.4 (102/1595)	9.5 (75/787)	10.7 (17/159)
**Personal service staff**	3.9 (20/517)	1.8 (29/1595)	1.0 (8/787)	5.0 (8/159)
**Ambulance staff**	3.5 (18/517)	3.5 (56/1595)	5.5 (43/787)	4.4 (7/159)
**Other**	12.4 (64/517)	11.9 (190/1595)	10.5 (83/787)	8.8 (14/159)
Occupation settings				
**Hospital**	62.5 (323/517)	57.6 (919/1595)	60.0 (472/787)	56.6 (90/159)
**Community or Private Practice**	21.3 (110/517)	30.3 (483/1595)	33.7 (265/787)	20.1 (32/159)
**Residential aged or disability care facility**	16.2 (84/517)	12.1 (193/1595)	6.4 (50/787)	23.3 (37/159)
Employment status				
**Full-time**	35.6 (170/477)	41.1 (634/1541)	45.1 (344/763)	36.6 (53/145)
**Part-time**	64.4 (307/477)	58.9 (907/1541)	54.9 (419/763)	63.4 (92/145)
Regionality				
**Regional**	30.2 (121/401)	24.7 (362/1463)	21.7 (158/729)	30.7 (39/127)
**Metropolitan**	69.8 (280/401)	75.3 (1101/1463)	78.3 (571/729)	69.3 (88/127)
Any comorbidity				
**Yes**	33.4 (143/428)	33.5 (477/1424)	33.9 (239/704)	22.7 (30/132)
**No**	66.6 (285/428)	66.5 (947/1424)	66.1 (465/704)	77.3 (102/132)
Has been tested for COVID				
**Yes**	71.1 (366/515)	80.4 (1274/1585)	81.0 (637/786)	77.1 (121/157)
**No**	28.9 (149/515)	19.6 (311/1585)	19.0 (149/786)	22.9 (36/157)
